# Recalibration of myoelectric control with active learning

**DOI:** 10.3389/fnbot.2022.1061201

**Published:** 2022-12-15

**Authors:** Katarzyna Szymaniak, Agamemnon Krasoulis, Kianoush Nazarpour

**Affiliations:** ^1^Edinburgh Neuroprosthetics Laboratory, School of Informatics, The University of Edinburgh, Edinburgh, United Kingdom; ^2^School of Engineering, Newcastle University, Newcastle-upon-Tyne, United Kingdom

**Keywords:** myoelectric control, active learning, machine learning, prosthetics, adaptation

## Abstract

**Introduction:**

Improving the robustness of myoelectric control to work over many months without the need for recalibration could reduce prosthesis abandonment. Current approaches rely on *post-hoc* error detection to verify the certainty of a decoder's prediction using predefined threshold value. Since the decoder is fixed, the performance decline over time is inevitable. Other approaches such as supervised recalibration and unsupervised self-recalibration entail limitations in scaling up and computational resources. The objective of this paper is to study active learning as a scalable, human-in-the-loop framework, to improve the robustness of myoelectric control.

**Method:**

Active learning and linear discriminate analysis methods were used to create an iterative learning process, to modify decision boundaries based on changes in the data. We simulated a real-time scenario. We exploited least confidence, smallest margin and entropy reduction sampling strategies in single and batch-mode sample selection. Optimal batch-mode sampling was considered using ranked batch-mode active learning.

**Results:**

With only 3.2 min of data carefully selected by the active learner, the decoder outperforms random sampling by 4–5 and ~2% for able-bodied and people with limb difference, respectively. We observed active learning strategies to systematically and significantly enhance the decoders adaptation while optimizing the amount of training data on a class-specific basis. Smallest margin and least confidence uncertainty were shown to be the most supreme.

**Discussion:**

We introduce for the first time active learning framework for long term adaptation in myoelectric control. This study simulates closed-loop environment in an offline manner and proposes a pipeline for future real-time deployment.

## 1. Introduction

Benefiting from an exponential increase in computational power and the availability of data, machine learning stands as one of the main pillars of the digital revolution. Not surprisingly, the literature on myoelectric control has also seen the reemergence of academic interest in the use of machine learning for the classification of the myoelectric signals (Buongiorno et al., 2019). Likewise, myoelectric industry has recently developed or adopted machine learning-based solutions, although large scale deployment of such methods is challenging because the performance of current machine learning algorithms degrades over time (Kaufmann et al., 2010; Amsüss et al., 2013; He et al., 2013). It is caused by a range of intrinsic factors, e.g., changes in muscle physiology (atrophy or hypertrophy; Kyranou and Erden, 2018) and motor behavior (Hahne et al., 2020) as well as extrinsic perturbations such as electrode displacement and arm position (Scheme and Englehart, 2011; Radmand et al., 2014).

A conventional way to enhance the robustness of machine learning-based decoding is to use a *post-hoc* error detection system that determines whether a decoder's prediction is certain enough. Based on predefined criteria of certainty, the model outputs a movement class label, and the prosthesis executes the movement if the predicted probability attains a preset level of confidence (Scheme et al., 2013; Amsüss et al., 2014; Al-Timemy et al., 2018; Krasoulis et al., 2020). In this approach, the classifier is fixed, that is, it is not updated upon the detection and rejection of errors. Hence, these approaches do not address, but partially circumvent, the issue of performance decline over time. To address this concern, ideally both human motor function as well as the machine learning system should modify their behavior over time, that is the so-called co-adaptation. However, in all earlier literature that advocates for motor learning/adaptation for prosthesis control (Radhakrishnan et al., 2008; Segil and Weir, 2015; Dyson et al., 2018, 2020; Antuvan, 2019; Segil et al., 2020), a fixed decoder was used and learning/adaptation was quantified by probing the human's motor behavior. On the other hand, despite the use of terms such as co-adaptive, machine adaptation has been shown to work for short term data and in fixed environment, without any direct involvement from the user beyond the operation of the interface (Hahne et al., 2015; Vidovic et al., 2016; Igual et al., 2019).

To the best of our knowledge there is no platform that enables and explicitly verifies a human user and the machine co-adapt. Nevertheless, research on the development of closed-loop frameworks that allow machine adaptation is ongoing. Broadly, these can be grouped into supervised recalibration and unsupervised self-recalibration.

In *supervised re-calibration*, a set of predefined tasks is performed at every iteration of decoder update (He et al., 2015). This naive approach puts the onerous of regular system updates on a user. An alternative approach uses a new set of labeled samples (Liu et al., 2014; Vidovic et al., 2015). Specifically, Liu et al. (2014) modified the classifier using all prior models from previous days to recalibrate the decoder. In contrast, Vidovic et al. (2015) focused on optimizing the decoder using small runs of daily data. Examples of periodic recalibration using transfer learning were also used in an attempt to decrease the amount of needed data (Prahm et al., 2017; Côté-Allard et al., 2019). Moreover, Gu et al. (2018) used an incremental learning scheme to redefine a representative sample set for model training. These scenarios however can account for small shifts in the distribution of the data only, and hence are more suited for short term laboratory research.

System recalibration shows performance improvement, but retraining is not a sustainable solution for long-term prosthesis usage. It disrupts the prosthesis use, requires memory, and is costly computationally. To address these shortcomings, the method of pseudo-labeling for *unsupervised self-calibrating* has been proposed. By using models' predictions to annotate/label the data, the system is able to adapt using only estimation of the user intents (Sensinger et al., 2009; Chen et al., 2013; Zhai et al., 2017; Côté-Allard et al., 2020). Given its unsupervised nature, it removes the burden of successive retraining processes and hence becomes easier for the user. Initial attempts toward system recalibration involved *post-hoc* comparison analysis, by enlarging the training pool with respect to controller confidence using unsupervised entropy-based confidence threshold (Sensinger et al., 2009). Using the predictions of testing data from previous sessions, Chen et al. (2013) updated the model to maintain the performance. Such a self-enhancing classifier is a self-recalibrating system because it continuously updates its parameters without enlarging the dataset, that is the testing data is discarded after each model update. Preliminary results were promising, but they were based on single-day data acquisition (with 2–3 and 6–7 h time span between training and testing data collection). Recently, Zhai et al. (2017) suggested refining decoder by using the predictions from the previous training sessions and those based on adjacent window segments. This concept was based on assumption that neighboring segments share the same class movement. To enhance relabeling using context-based predictions, domain adaptation was applied in Côté-Allard et al. (2020). They report performance improvement over the no calibration setting. Yet, when compared with recalibration, the performance degradation was significant.

The aim of this paper is to demonstrate, in an offline setting, this bottleneck can be addressed fundamentally using active learning (Settles, 2009). We offer a structured perspective and methodology, *via* which a user can interact with the machine. The intuition behind this approach is based on selecting new (unlabeled) data samples that are representative of the underlying real-world data distribution or they provide new information for the model during the training process. Specifically, the objective of active learning algorithms is to define samples for optimal model training by minimizing labeling costs while maximizing a model's performance.

We will argue that this approach can provide a sustainable solution for the shortcomings of all previously stated approaches. Active learning is capable of detecting new regions of interest and modifying decision boundaries based on changes in data (possible distribution shifts and others). Rejection-based calibration dismisses those samples. This is a feasible solution in a short term scenario, however over time it will lead to discrepancy and system limitations. This problem can be solved by simply retraining the decoder using more data (supervised recalibration). Yet, active learning increases robustness of the model by choosing informative samples and simultaneously using less samples for training (a.k.a. decreasing computational costs). Unsupervised calibration tries to tackle the problem from the annotation point of view. However, as it often relies on initial calibration being well-optimized, if impaired it can lead to a drastic decrease in the performance of the model over time. Active learning can optimize this performance with as fewest samples as possible, leaving this approach supreme in all aspects.

## 2. Materials and methods

### 2.1. Data collection

Data was collected as part of Krasoulis et al. (2020). For completeness we briefly described the experimental process. The myoelectric signals were collected from the forearm of 12 able-bodied and the stump of two subjects with trans-radial limb loss. See Table 1 for a description of participants with limb loss. Prior to the placement of myoelectric sensors, we cleansed participants' skin using 70% isopropyl alcohol. In the first group, we placed 16 Tringo^*TM*^ sensors (Delsys, USA) on the participants' in two rows of eight equidistant electrodes. For subjects with limb loss, we used 13 and 12 sensors based on their physiology of the stump after amputation. We used an adhesive elastic bandage to secure the locations of the electrodes throughout the sessions.

**Table 1:** Medical records of amputee subjects.

**Gender**	**Age**	**Type of amputation**	**Cause of amputation**	**Years**	**Missing limb**	**Hand dominance** ** (prior to amputation)**	**Prosthesis use**
Male	28	Transradial	Car accident	6	Right	Right	Split hook
Male	54	Transradial	Cancer (epitheliod sarcoma)	18	Right	Right	Split hook

Sampling rates in data recording were originally 1,111 and 128 Hz for the myoelectric channels and the inertial measurement data, respectively. This sampling rate was the standard and fixed hardware sampling rate of Delsys Trigno system when inertial data was recorded simultaneously. We did not use the recorded inertial data in this study. In the process of data cleaning, the Hampel filter was applied to up-sampled (2 kHz) data to eliminate the power line inference. This pre-processing followed the process introduced in Atzori et al. (2014). The data was then band-pass filtered in the range 30–400 Hz using a 4th-order Butterworth filter. In this paper, we used only the myoelectric data. In a sliding window of 128 ms (256 raw signal samples) with an increment of 50 ms (100 raw signal samples) the waveform length for each channel was calculated resulting in 16 values per window (one per sensor), and 13 and 12, respectively participants with limb loss. In the discussion, we described why we chose only one feature.

Data was collected in two identical sessions, namely *T*1 and *T*2, which were conducted sequentially on the same day. During the recording session, each subject was instructed to perform the grips that were shown on a computer screen. The experiment involved five grips, namely power grip, lateral grasp, tripod grasp, index pointer, and hand open. During two blocks of data recording, each grip was repeated 10 times, with 5 s of muscle activation followed by 3 s of rest.

The first dataset, *T*1, was used for training and the second dataset, *T*2, was used for testing the decoder.

### 2.2. Active learning

A general framework for an active learner includes two sets of data, namely an initial small set of labeled data *L* and a large pool of unlabeled samples *U*. Based on initial subset of data, L^*^ ⊂ *L*, a model *f*(*x*|L^*^) is trained. The aim of an active learner is to choose an L^*^ such that *f*(*x*|L^*^) ≈ *f*(*x*|*L*). Note that these two sets are not the same sets as *T*1 and *T*2.

**Figure 1:**
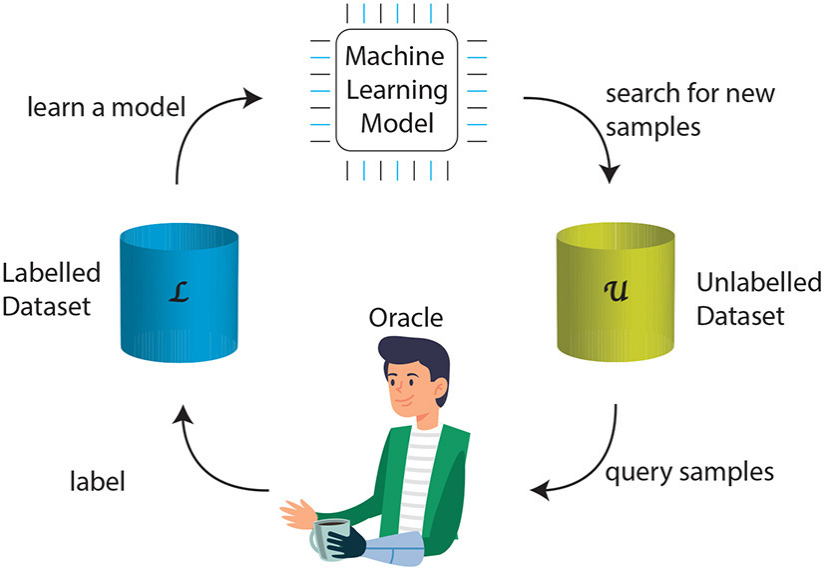
The generic framework of active learning. The feedback loop includes a small set of labeled data *L* that is used to train a machine learning model. Based on that, the active learner explores a large pool of unlabeled data *U* and selects new samples. These samples are then passed to an oracle, which would be a prosthesis user in this paper, for labeling. These samples join the training set for model retraining.

To obtain L^*^, the active learner adopts a query strategy and a current model *f*(*x*|*L*′), where *L*′ is a transitional annotated set, and selects new samples for annotation by an oracle, e.g., the prosthesis user. The oracle has the task of labeling the unlabeled data. Figure 1 presents a generic active learning framework. Starting from the initial dataset, the model is trained. Adopting on a query strategy, the active learner selects new instances *x*_*U*_ from the pool of unlabeled data for labeling by the oracle and moving them from *U* to *L*. The model is then updated.

There are three main strategies of sampling from the pool of unlabeled data, namely, stream-based selective sampling (Atlas et al., 1990), membership query synthesis (Angluin, 1988), and pool-based active learning (Lewis and Gale, 1994). We adopted the pool-based sampling method in this proof of concept, offline, study. We also shared our views as to how active learning can be implemented in a real-time myoelectric control setting.

### 2.3. Query strategy

Active learning is based on defining optimal criteria for deciding whether the oracle needs to label an unlabeled sample, that is a query. Using measures that determine the *informativeness* and/or *representativeness* of a sample, there are many algorithms for querying the label. Informativeness is typically measured with uncertainty sampling. In this method, the active learner finds samples in the pool about which the decoder is least certain. We used the following metrics:

1. *Least Confidence Uncertainty* (Lewis and Gale, 1994) considers querying a sample that it is the least certain about the predicted class:
  (1) $$x_{LC}^{*}=\underset{x}{\arg\;\max}\;1-P_{\theta }(\hat{y}|x)$$
  where $\hat{y}=\arg\;\max_{y}P_{\theta }(y|x)$. This approach is intuitive, however, it focuses on the most probable label without considering the remaining label distribution.
2. *Smallest Margin Uncertainty* (Scheffer et al., 2001) considers the uncertainty between two most likely labels:
  (2) $$\begin{array}{l} x_{M}^{*}=\underset{x}{arg min}\left[P_{\theta }\left(\hat{y_{1}}|x\right)-P_{\theta }\left(\hat{y_{2}}|x\right)\right] \end{array}$$
  where $\hat{y_{1}}$ and $\hat{y_{2}}$ are the two labels with the highest probabilities predicted by the model. Intuitively, large margins are easy to identify as they are expected to be far from the decision boundary. Instances with small margins dictate potential ambiguity in discriminating between the two classes. Acquiring such instances improves classification within the decision boundary area.
3. *Entropy Reduction* (Shannon, 1948) sampling strategy uses entropy to measure the amount of information necessary to depict a distribution. As such, often described in machine learning as a measure of impurity.
  (3) $$\begin{array}{l} x_{H}^{*}=\underset{x}{arg max}-\underset{i}{\sum }P_{\theta }\left(y_{i}|x\right)\log P_{\theta }\left(y_{i}|x\right) \end{array}$$
  where *y*_*i*_ indicates all plausible annotations.

Alternative to the single sample selection, a batch-mode active learning can be applied, which allows querying larger sets of samples. We used ranked batch-mode active learning (RBMAL) (Cardoso et al., 2017), which generates an optimized ranked list (*Q*) of unlabeled samples. Specifically, the uncertainty estimation step calculates two sets. First, uncertainty score *U*_*score*_ for all unlabeled samples in *U*, and *D*_*estimate*_ set with *L* samples and already included (if any) samples within the ranking *Q*. The set *D*_*estimate*_ presents the expected training set given all samples within the ranking *Q* will be annotated. Then, for instance, ranking score value for each sample within *U* (Equation 4) is calculated using similarity score Φ and *U*_*score*_. To define Φ, the highest similarity between samples from the expected training set *D*_*estimate*_ and each *U* instance was calculated. Combining this information enables the calculation of *score* to determine an instance with the highest value to be added to *Q* (and removed from *U*). This process for ranking construction is repeated until *U* = ∅ or |*Q*| = *batchSize*.


(4) $$\begin{array}{l} score\left(x\right)=\alpha \times \left[1.0-\text{Φ}\left(x,D_{estimate}\right)\right]+\left(1.0-\alpha \right)\times U_{score}\left(x\right), \end{array}$$

where $\alpha =\frac{\left|U\right|}{\left|U|+|L^{\prime }\right|}$ balances the trade-off between exploration and exploitation.

### 2.4. Experiment design

We ensured that all classes, including the rest and grasp classes, had the same number of samples, i.e., balanced classification. This was achieved by under-sampling of the rest class which appeared between each grip repetition. The initial training set *L* ⊂ *U* included 300 samples in total, that is 50 samples per class. We sought to simulate a real-life scenario and hence picked the first 50 samples of data in each class in *T*1. This is equivalent to ~2.6 s of myoelectric data per class, in the time domain. We treated the rest of the data in *U* as unlabeled samples. All training data was from the first recording session, *T*1.

For single instance sample selection we compared the four approaches, namely random sampling (i.e., passive learning), least confidence uncertainty, smallest margin uncertainty, and entropy reduction. We used the results of the random sampling approach as benchmark. We made 1,500 queries iteratively and at each iteration the training set was normalized to its *z*-scores. Then a linear discriminant analysis model was fitted on the normalized training set, *L*′. All resultants 1,500 models were tested with the data from the second session, *T*2.

The second analysis compared the performance of single instance sampling with that of two batch-mode sampling methods, namely naive and ranked. The former approach focuses on finding *n* best samples to query within single iteration. The latter learns to obtain an optimal set of samples to query from. The ranked method removes potential sample redundancy within a queried batch (if queried samples are too similar) by emphasizing sample diversification using the Euclidean distance measure.

To avoid any bias in training the decoder using the random batch sampling method, we imposed the constraint of sampling one sample per class within the same batch, keeping the batch size to 6. This ensured an independent and identically distributed random sampling condition, imposed on random sampling scenario, throughout all the iterations. All previously mentioned query strategies were used in this analysis too.

### 2.5. Analysis

Metrics used for the evaluation of the proposed approaches included accuracy and F1-score (i.e., harmonic mean of precision and recall). Accuracy is the percentage of samples that have their labels correctly recognized. F1-score was calculated per class using one-vs.-all scheme and then averaged across the classes, that is micro-averaging. These measures, together, reflect the overall success of the proposed approach.

## 3. Results

### 3.1. Single query sampling

**Figure 2:**
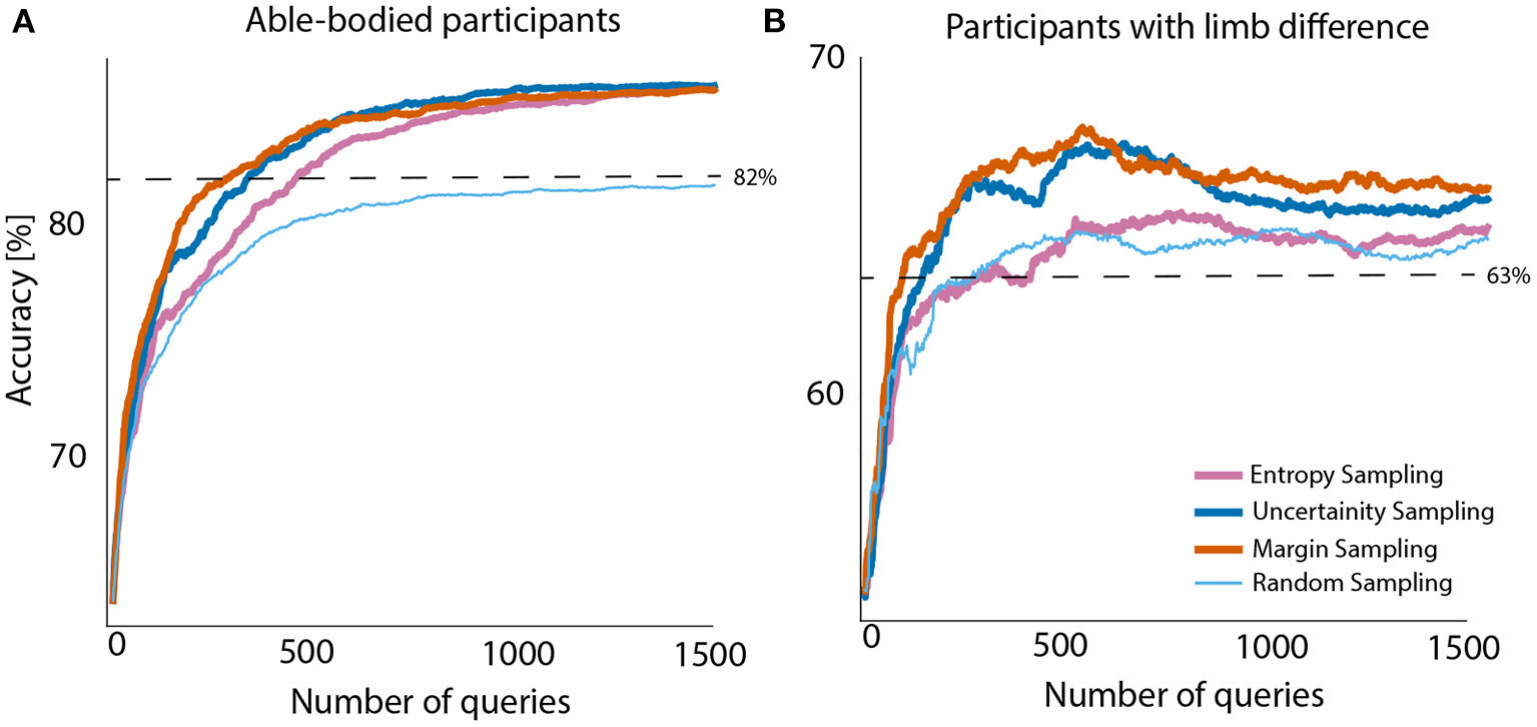
Averaged test accuracy across all able-bodied participants **(A)** and people with limb difference **(B)**. Initial training sets for subjects in both groups contained 50 samples from each class. Initial accuracy without active learning is presented at query step zero. Offline baseline: 82% **(A)** and 63% **(B)** for able-bodied and people with limb loss, respectively.

Four query strategies were compared across 1,500 single queries (equivalent to 3.2 min of data). Figure 2 presents the accuracy scores of all query strategies plotted against a number of queried samples and averaged across all participants. The classifier was initially trained with 50 samples from each class (giving 15.9 s of training data per subject). The mean accuracy and standard deviation for able-bodied participants and people with limb difference, before the first query was 63.66 ± 16.13 and 54.24 ± 2.65%, respectively.

Analysis of data from able-bodied participants showed that, active learning, irrespective of the adopted sampling method, outperforms random sampling by 4–5%. This improvement for people with limb difference was ~2%. Although entropy sampling outperformed the baseline, it required more samples to level off with remaining active learners. These improvements might seem small, but we would like to remind the reader that they were achieved with only 1,500 samples (only 3.2 min of data) and very little additional computations. For completeness we also considered the case of 200 queries only. Details on obtained results for both query numbers and all sampling strategies are presented in Table 2.

**Table 2:** Method comparison between query 200 and 1,500. All accuracy are reported in %.

	**Able-bodied**				**Amputee 1**				**Amputee 2**			
	**Q200**		**Q1500**		**Q200**		**Q1500**		**Q200**		**Q1500**	
	**Acc (std)**	**F1**	**Acc (std)**	**F1**	**Acc**	**F1**	**Acc**	**F1**	**Acc**	**F1**	**Acc**	**F1**
Entropy	77.2 (12.7)	0.76	85.9 (7.3)	0.86	65.1	0.65	67.8	0.66	61.1	0.58	62.3	0.59
Uncertainty	79.1 (11.8)	0.78	86.1 (7.3)	0.86	68.4	0.67	68.1	0.66	62.1	0.58	63.7	0.61
Margin	80.9 (10.9)	0.8	85.9 (7.6)	0.86	69.6	0.68	67.8	0.66	61.6	0.58	64.7	0.62
Random	76.7 (11.9)	0.76	81.7 (9.1)	0.82	66.8	0.66	67	0.65	60.1	0.58	62.4	0.6

### 3.2. Comparison of queries

**Figure 3:**
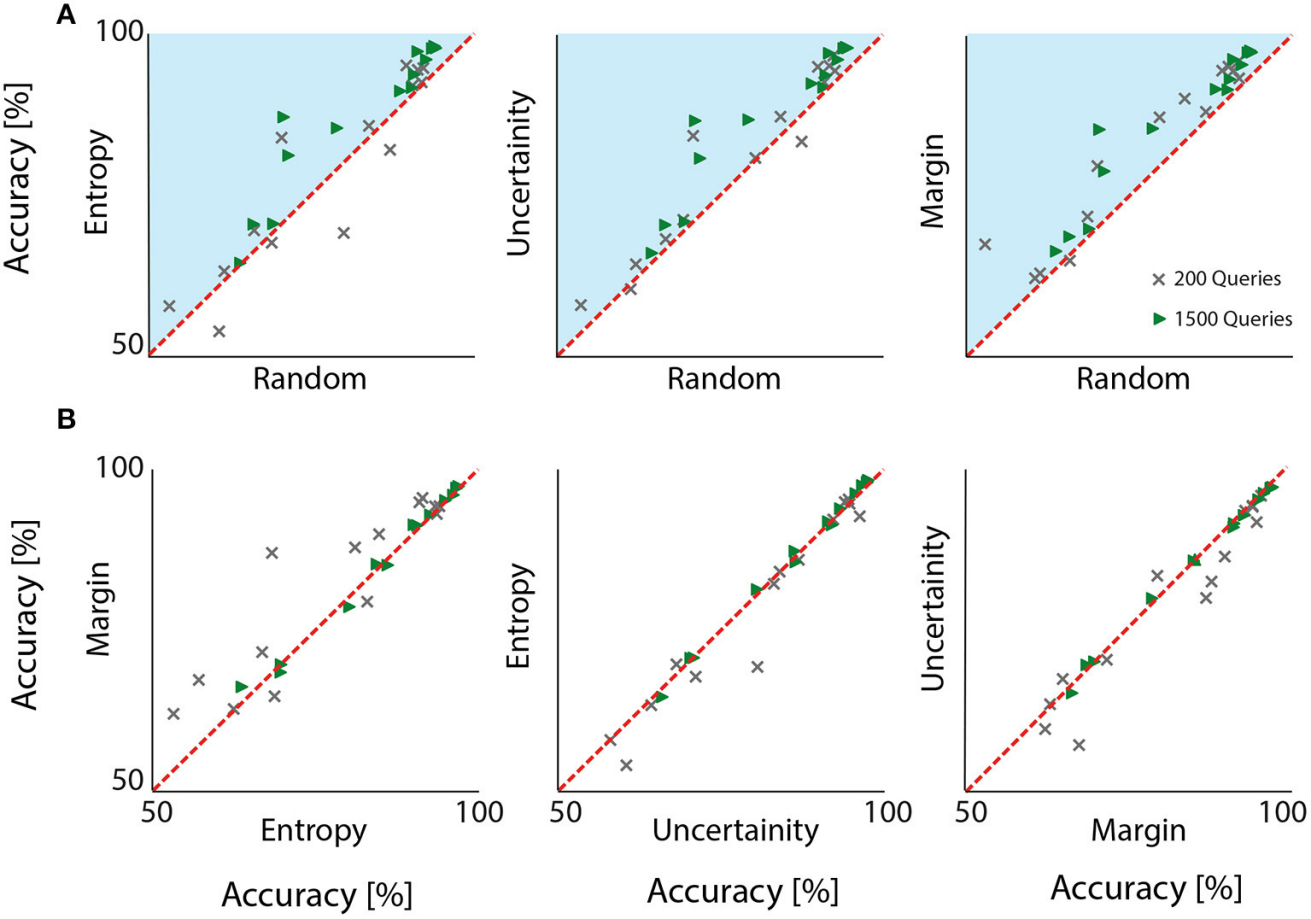
Accuracy plots to compare all investigated query strategies at two stages: After the first 200 and after all 1,500 queried samples. Each marker represents results from one participant. Random sampling (in **A**) compared against the remaining three approaches shows a visible tendency to achieve lower accuracy. Irrespective of the choice of query sampling, active learning outperformed the conventional random sampling, because results from all participants lie above the unity line, i.e., in the blue zone, when 1,500 samples were selected. A similar conclusion can be drawn when 200 samples wee drawn although for a very few participants, random sampling was more successful. When different active learning query strategies were compared **(B)**, different query sampling were comparable but margin sampling seemed to offer a lightly higher performance.

Figure 3 depicts accuracy scores obtained by all the subjects after querying the first 200 and after all 1,500 samples. For a comprehensive analysis, all combinations are reported. In the first row, three adjacent plots map out random sampling against active learning query strategies. In all cases, active learning outperformed random sampling in almost all subjects, that is markers representing accuracy results from individual participants lie above the unity line in Figure 3A. When active learning approaches were compared against each other, results were relatively similar (c.f. Table 1). Interestingly, for most subjects, margin sampling proved slightly more successful.

**Figure 4:**
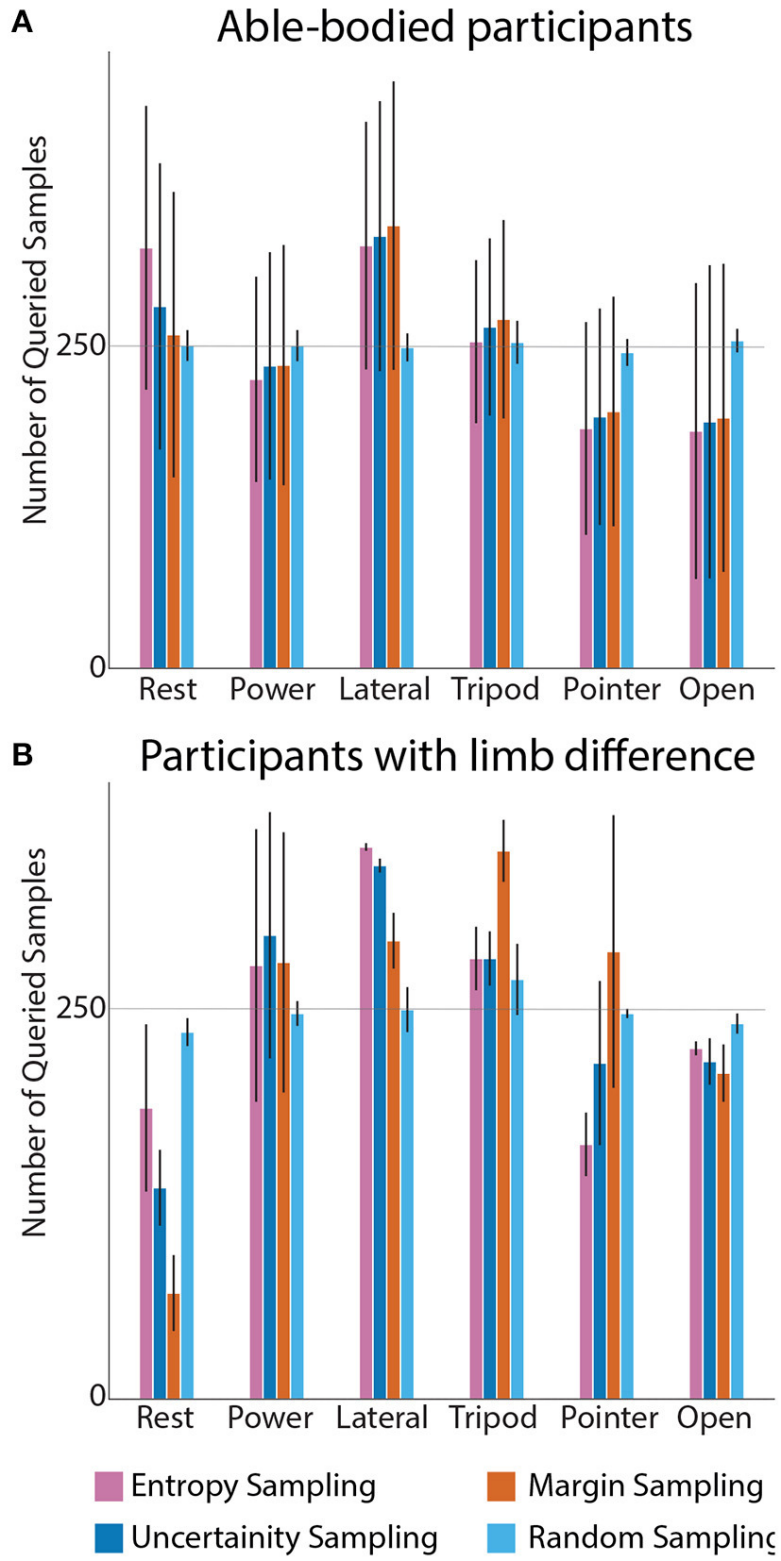
Grip instances acquisition during 1,500 querying process across all the subjects: able-bodied participants **(A)** and people with limb difference **(B)**. Query strategies are compared against class. In **(A)**, the highest demand for new instances within 1,500 queries was for the lateral grip. The pointer grip and hand open classes needed the fewest samples. In contrast to able-bodied participants, active learning requested fewest samples for the rest class. In all cases, random sampling drew 250 samples on average.

Figure 4 represents number of queried instances from each grasp per query strategy (Figure 4A), averaged across all able-bodied participants. In random sampling, the data is picked with discrete and uniform probability distribution across all the classes, hence when 1,500 samples is being queried, ~250 samples is acquired from each grip class. These are then averaged across all participants. However, with active learning, we can observe a class-specific sampling. When inspecting grip trends for active learning query strategies, we observed rest class being acquired the most by entropy sampling for both participant groups. Characteristic for all strategies, most samples were queried for the lateral grasp class. This is explainable from an anatomical point of view: the abductor pollicis brevis longus muscle, which is responsible for thumb movement and stabilization, is a deep muscle in the forearm. Hence the myoelectric data from this muscle is affected significantly by volume conduction and interference from other muscles. As such active learning prioritized querying this class.

Figure 4B shows the same analysis on data, averaged across the two people with limb difference. The most striking difference between data from the two groups was that active learning required significantly smaller number of samples for the rest class. Yet, the inter-subject differences were too large to warrant statistical significance.

### 3.3. Batch mode

For batch mode analysis, two scenarios were considered: naive batch sampling and ranked batch sampling. Both approaches followed similar protocol for experimental design as in single instance sampling. Active learning framework was constructed with a linear discriminator analysis decoder, 50 initial samples (acquired in the same manner as in the previous experiment) and 250 queries (with six samples per batch, corresponding to 3 ms) which is the equivalent of the number of samples obtained after 1,500 queries for single instance sampling (250 queries × 6 samples).

**Figure 5:**
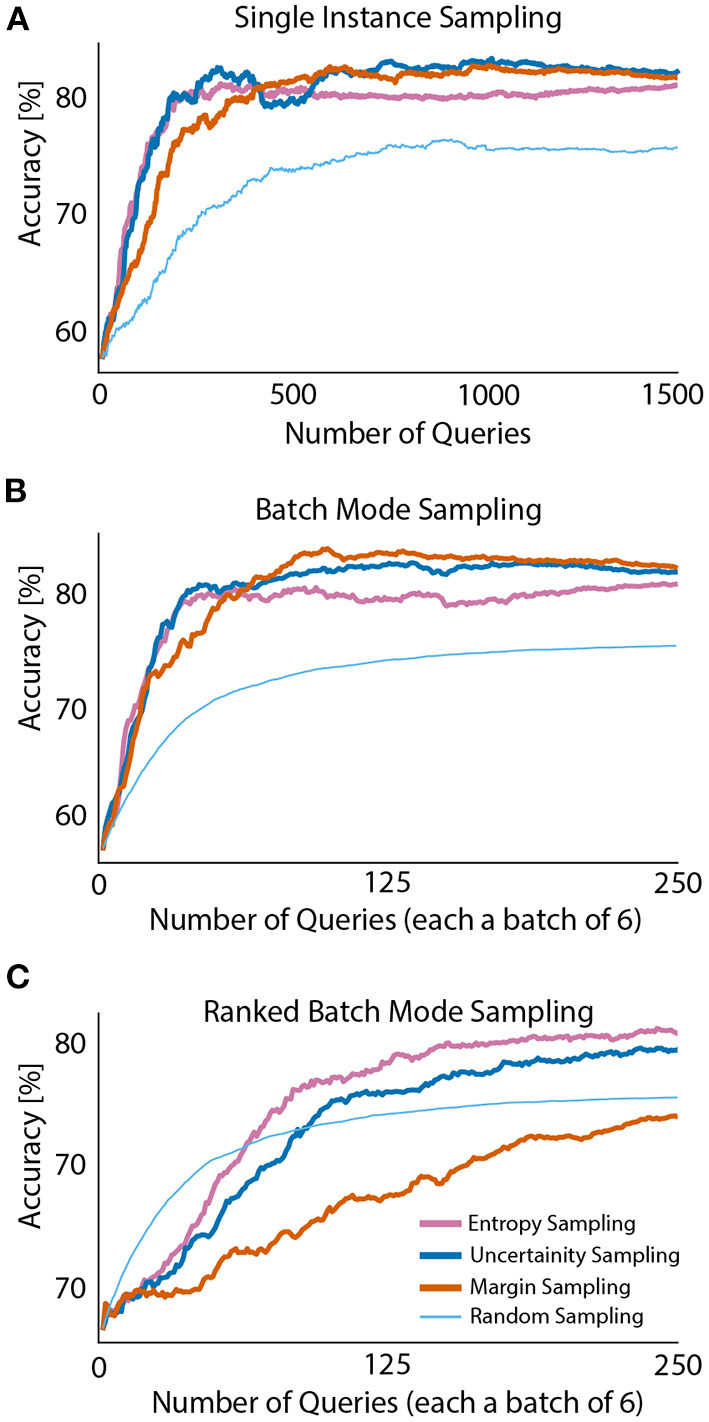
Comparison of single instance **(A)**, batch mode **(B)** and ranked batch mode **(C)** sampling methods in one representative participant.

Observed results in Figure 5 indicate single instance sampling and batch mode sampling behave in a similar manner. Margin and uncertainty sampling perform the best. However, ranked batch mode sampling presents divergent results. In the initial stages of querying, random batch sampling outperformed active learning query strategies. Entropy and uncertainty sampling slowly converged and outperformed random batch sampling (as the number of iterations increased). Important to note, random batch sampling was constraint to querying a sample from each class (within a single batch) at each iteration to ensure no bias across classes occurs in further iterations. Based on this constrained, batch size for sampling new instances was set to six (number of classes) for all query approaches. In contrast with the previous approaches, margin ranked batch sampling had the lowest performance across all sampling methods.

## 4. Concluding remarks

We introduced active learning for myoelectric control as a potential approach to ease re-calibration and enhance long-term decoding stability. We reported the results of an offline, feasibility study in a simulated human-in-the-loop setting. We will draw conclusions as to how the proposed approach can be implemented in real-time. We envisage that the machine learning decoder, supplied with only a small subset of labeled data, using an active learning boost can efficiently exploit an abundance of unlabeled data in closed-loop, real-life settings.

We utilized a linear decoder, as an example. Nevertheless, we could have used active learning with any machine learning decoder. Results reported during the experiments prove the effectiveness of this approach with such a simple decoder. The input feature was the waveform length of the myoelectric signals. We chose it because it has been shown as one of the most effective features for myoelectric signal classification. This approach also enabled us to avoid the curse of dimensionality, especially in the initial phase of learning when the decoder was trained on the 300 samples only.

Within a pool-based setting, three sampling methods were considered: single instance sample selection, batch-mode and ranked batch-mode. Although, active learning can consider a cold start with no initial dataset, using a small number of labeled samples can initially boost active learning performance. We included the first 50 samples from each class, equivalent to 2.65 s of data, mimicking real-life scenarios.

We demonstrated that active learning outperforms random sampling. Within proposed query strategies, the smallest margin sampling and least confidence uncertainty achieved higher accuracy in most participants. Intuitively, we expected large margins to be further away from the decision boundary, which consequently means instances with a small margin could potentially provide information to improve the decision boundary. Least confidence uncertainty focuses on sampling cases which the model is the most unsure about. In the initial stages of training, entropy sampling presents a decrease in performance for able-bodied participants and is comparable to baseline results for amputees. This type of behavior can be caused by emphasizing feature space exploration over exploitation by query strategy.

As stated earlier, the objective of an active learner is to optimize amount of data needed for training the decoder while maximizing its performance. Using passive learning, i.e., random sampling, gave us performance comparison against the baseline. Since our experiment is static, that is the pool of the data does not change with time, the active learners after querying all the samples will converge to the same performance as the random sampling solution. This would provide the results equivalent to offline training on the entire block *T*1 (initial and pool data set altogether). Figure 2 presents the results after training the decoder on the entire block *T*1. As noticed, the performance of active learner at query no. 1500 is greater than when entire data set is being used. We presume the potential noise of the signal and transient periods created noisy input data, leading to decreased performance.

When considering query sampling size, we applied batch-mode active learning and ranked batch mode active learning. Performances of single instance sampling and BMAL were comparable. In RBMAL, exploration (uncertainty measure) was compromised by exploitation (distance measure). Thus, active learning at the initial querying stages showed a lower performance than the benchmark. Among those, margin sampling was the one to perform the worst. Smallest margin sampling looks for *n* best samples that potentially lay on decision boundaries (difference between two most probable classes is the smallest in respect to the pool of the data). We presume this can be potentially caused by samples with the smallest margin being quite similar/close to each other which is compromised by similarity score (distance measure) to avoid potentially redundant samples.

### 4.1. From offline to real-time deployment

**Figure 6:**
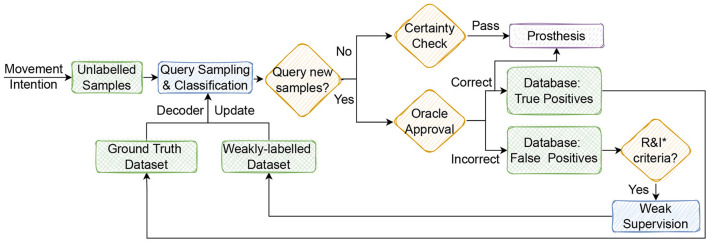
The envisaged real-time operation of a user-in-the loop setting with an active learning. Movement intention generates new signal data each second (w.r.t. the sampling rate). Unlabeled samples after being preprocessed are classified by pre-trained decoder. Active learner using query sampling criteria decides whether to request the label or not. Confident samples pass certainty check criteria to be sent to the prosthesis actuator. If a sample is queried by the active learner, the oracle dis-/approves the decoder prediction. Incorrect predictions are filtered with R&I (representativeness and informativeness criteria) for outlier detection and *interesting* samples to be weakly-labeled. These combined with ground truth samples are used to update the decoder. *R&I, representativeness and informativeness.

Creating a human-in-the-loop environment has the potential to motivate the users to engage more with the decoder and ultimately lead to an enhanced control of the prosthesis. We presented a closed-loop setting in which an oracle and a prostheses user are the same. Figure 6 offers one perspective by which an active learning paradigm could be implemented in real-time.

One might assume that asking the user for specific labels in a real-time setting, e.g., in the stream-based active learning paradigm, can prove difficult and indeed no hardware setting is readily available to capture such user input. To minimize user input when the active learner is requesting incoming data to be labeled, the user has to specify only whether the decoder prediction was correct or not (a binary choice). Real-time prosthesis movement is performed only after unlabeled samples from movement intention pass safety check (certainty/confidence criteria) or the user has confirmed the decoder prediction. The additional *R&I* criteria on incorrect predictions (false positives) would work as a filter for noise/outlier detection and sample re-selection for weak supervision.

This way samples will augment additional information and can be re-integrated into the pool. Furthermore, this approach has the potential to minimize the annotation/labeling noise. It is possible that additional steps may be required to resolve issues around the user bias.

### 4.2. Limitations

A limitation of this work is in using a simulated human annotator as an oracle for the active learning framework; meaning the entire dataset contains pairs of {*x, y*} data. Nonetheless, labels were introduced to the learner only when requested by query strategy.

In this study, we focused on the informativeness of the samples. Further experiments with alternative query strategies may be needed for more detailed understanding of the potential benefits of active learning for prosthetic control. Considering long-term adaptation of the prosthesis in the real-world, the exploration versus exploitation paradigm could be further investigated. The trade-off between data informativeness and representativeness could potentially enhance the decoder functionality. While initially greedy feature space exploration enables the model to rapidly boost its performance, with time defining specific grip characteristics could possibly establish its singularity aspects.

Data used during this study presented an example of a high-quality dataset with multiple subjects including people with limb differences. From each participant, data was recorded in two distinct, but consecutive, sessions in 1 day. Between the two sessions, the electrodes remained stuck to the skin with the help of adhesive tape. This arrangement minimized the between-session variability in the data, as one would expect to see with dry electrodes especially when considering the donning and doffing of a prosthesis socket. This is neither a limitation of the proposed active learning method nor a shortcoming of the paper. Nevertheless, this repeatable recording method minimized the additional benefit of using active learning.

Commercial myoelectric controllers such as Sense (IBT, USA), Myo Plus (Ottobock, Germany), and CoAPT Gen2 (CoAPT, USA) provide supervised recalibration options with varying levels of user-friendliness. CoAPT also offers Adaptive Advance™as an automatic, continuous-learning algorithm that combines new calibration data into the existing state of users' control. However, no technical information about this adaptive calibration method is available, beyond the generic text in paragraph [0011] of CoAPT pattern recognition patent (WO2020172261).

## 5. Conclusions

We explored the feasibility of using active learning for pattern recognition-based myoelectric control. We observed that all adopted active learning strategies improve decoders adaptation and significantly reduce or optimize the amount of training data on a class-specific basis. Query strategies, such as smallest margin sampling and least confidence uncertainty were found to improve decoders performance when compared to conventional random sampling. Future work will include the development of a setup for testing the active learning in real-time and with a prosthesis user in the loop.

## Data availability statement

The raw data supporting the conclusions of this article will be made available by the authors, without undue reservation.

## Ethics statement

All experimental procedures were in accordance with the Declaration of Helsinki and approved by the local Ethics Committees of the School of Informatics, University of Edinburgh (#201507160854) and School of Engineering, Newcastle University (#14-NAZ- 056). All participants read an information sheet and gave written informed consent prior to the experimental sessions.

## Author contributions

KS and KN conceived the project, designed the experiment, analyzed the data, and wrote the first draft of the manuscript. AK collected the data. All authors contributed to the manuscript. All authors contributed to the article and approved the submitted version.

## References

[B1] Al-Timemy A. H. Bugmann G. Escudero J. (2018). Adaptive windowing framework for surface electromyogram-based pattern recognition system for transradial amputees. Sensors 18:2402. 10.3390/s18082402 30042296 PMC6112043

[B2] Amsüss S. Goebel P. M. Jiang N. Graimann B. Paredes L. Farina D. (2014). Self-correcting pattern recognition system of surface EMG signals for upper limb prosthesis control. IEEE Trans. Biomed. Eng. 61, 1167–1176. 10.1109/TBME.2013.2296274 24658241

[B3] Amsüss S. Paredes L. P. Rudigkeit N. Graimann B. Herrmann M. J. Farina D. (2013). “Long term stability of surface EMG pattern classification for prosthetic control,” in 2013 35th Annual International Conference of the IEEE Engineering in Medicine and Biology Society (EMBC), 3622–3625. IEEE. 10.1109/EMBC.2013.6610327 24110514

[B4] Angluin D. (1988). Queries and concept learning. Mach. Learn. 2, 319–342. 10.1007/BF00116828

[B5] Antuvan C. W. (2019). Decoding human motion intention using myoelectric signals for assistive technologies (Ph.D. thesis). Nanyang Technological University, Singapore, Singapore.

[B6] Atlas L. David C. Ladner R. (1990). “Training connectionist networks with queries and selective sampling,” in Advances in Neural Information Processing Systems (Denver, CO: Citeseer), 566–573.

[B7] Atzori M. Gijsberts A. Castellini C. Caputo B. Mittaz Hager A. Elsig S.. (2014). Electromyography data for non-invasive naturally-controlled robotic hand prostheses. Sci. Data 1:140053. 10.1038/sdata.2014.53 25977804 PMC4421935

[B8] Buongiorno D. Cascarano G. D. Brunetti A. B. D. F. B. (2019). “A survey on deep learning in electromyographic signal analysis,” in International Conference on Intelligent Computing, eds D. S. Huang, Z. K. Huang, and A. Hussain (Springer International Publishing), 751–761. 10.1007/978-3-030-26766-7_68

[B9] Cardoso T. N. Silva R. M. Canuto S. Moro M. M. Gonçalves M. A. (2017). Ranked batch-mode active learning. Inform. Sci. 379, 313–337. 10.1016/j.ins.2016.10.037

[B10] Chen X. Zhang D. Zhu X. (2013). Application of a self-enhancing classification method to electromyography pattern recognition for multifunctional prosthesis control. J. Neuroeng. Rehabil. 10, 1–13. 10.1186/1743-0003-10-44 23634939 PMC3689085

[B11] Côté-Allard U. Gagnon-Turcotte G. Phinyomark A. Glette K. Scheme E. Laviolette F.. (2019). Virtual reality to study the gap between offline and real-time EMG-based gesture recognition. arXiv preprint. 10.1109/TNSRE.2021.3059741 33591919

[B12] Côté-Allard U. Gagnon-Turcotte G. Phinyomark A. Glette K. Scheme E. J. Laviolette F.. (2020). Unsupervised domain adversarial self-calibration for electromyography-based gesture recognition. IEEE Access 8, 177941–177955. 10.1109/ACCESS.2020.3027497

[B13] Dyson M. Barnes J. Nazarpour K. (2018). Myoelectric control with abstract decoders. J. Neural Eng. 15:056003. 10.1088/1741-2552/aacbfe 29893720

[B14] Dyson M. Dupan S. Jones H. Nazarpour K. (2020). Learning, generalization, and scalability of abstract myoelectric control. IEEE Trans. Neural Syst. Rehabil. Eng. 28, 1539–1547. 10.1109/TNSRE.2020.3000310 32634092

[B15] Gu Y. Yang D. Huang Q. Yang W. Liu H. (2018). Robust EMG pattern recognition in the presence of confounding factors: features, classifiers and adaptive learning. Expert Syst. Appl. 96, 208–217. 10.1016/j.eswa.2017.11.049

[B16] Hahne J. M. Dähne S. Hwang H.-J. Müller K.-R. Parra L. C. (2015). Concurrent adaptation of human and machine improves simultaneous and proportional myoelectric control. IEEE Trans. Neural Syst. Rehabil. Eng. 23, 618–627. 10.1109/TNSRE.2015.2401134 25680209

[B17] Hahne J. M. Wilke M. A. Koppe M. Farina D. Schilling A. F. (2020). Longitudinal case study of regression-based hand prosthesis control in daily life. Front. Neurosci. 14:600. 10.3389/fnins.2020.00600 32636734 PMC7318897

[B18] He J. Zhang D. Jiang N. Sheng X. Farina D. Zhu X. (2015). User adaptation in long-term, open-loop myoelectric training: implications for EMG pattern recognition in prosthesis control. J. Neural Eng. 12:046005. 10.1088/1741-2560/12/4/046005 26028132

[B19] He J. Zhang D. Sheng X. Zhu X. (2013). “Effects of long-term myoelectric signals on pattern recognition,” in International Conference on Intelligent Robotics and Applications, eds J. Lee, M. Lee, C. Liu, and J. H. Ryu (Berlin; Heidelberg: Springer), 396–404. 10.1007/978-3-642-40852-6_40

[B20] Igual C. Igual J. Hahne J. M. Parra L. C. (2019). Adaptive auto-regressive proportional myoelectric control. IEEE Trans. Neural Syst. Rehabil. Eng. 27, 314–322. 10.1109/TNSRE.2019.2894464 30676969

[B21] Kaufmann P. Englehart K. Platzner M. (2010). “Fluctuating EMG signals: investigating long-term effects of pattern matching algorithms,” in 2010 Annual International Conference of the IEEE Engineering in Medicine and Biology (Buenos Aires), 6357–6360. 10.1109/IEMBS.2010.5627288 21096692

[B22] Krasoulis A. Vijayakumar S. Nazarpour K. (2020). Multi-grip classification-based prosthesis control with two EMG-IMU sensors. IEEE Trans. Neural Syst. Rehabil. Eng. 28, 508–518. 10.1109/TNSRE.2019.2959243 31841413

[B23] Kyranou I. Erden S. V. M. S. (2018). Causes of performance degradation in non-invasive electromyographic pattern recognition in upper limb prostheses. Front. Neurorobot. 12:58. 10.3389/fnbot.2018.00058 30297994 PMC6160857

[B24] Lewis D. D. Gale W. A. (1994). “A sequential algorithm for training text classifiers,” in SIGIR'94 (Dublin: Springer), 3–12. 10.1007/978-1-4471-2099-5_1

[B25] Liu J. Sheng X. Zhang D. He J. Zhu X. (2014). Reduced daily recalibration of myoelectric prosthesis classifiers based on domain adaptation. IEEE J. Biomed. Health Inform. 20, 166–176. 10.1109/JBHI.2014.2380454 25532196

[B26] Prahm C. Paassen B. Schulz A. Hammer B. Aszmann O. (2017). “Transfer learning for rapid re-calibration of a myoelectric prosthesis after electrode shift,” in Converging Clinical and Engineering Research on Neurorehabilitation II (Segovia), 153–157. 10.1007/978-3-319-46669-9_28

[B27] Radhakrishnan S. M. Baker S. N. Jackson A. (2008). Learning a novel myoelectric-controlled interface task. J. Neurophysiol. 100, 2397–2408. 10.1152/jn.90614.2008 18667540 PMC2576223

[B28] Radmand A. Scheme E. Englehart K. (2014). On the suitability of integrating accelerometry data with electromyography signals for resolving the effect of changes in limb position during dynamic limb movement. J. Prosthet. Orthot. 26, 185–193. 10.1097/JPO.0000000000000041

[B29] Scheffer T. Decomain C. Wrobel S. (2001). “Active hidden Markov models for information extraction,” in International Symposium on Intelligent Data Analysis (Cascais: Springer), 309–318. 10.1007/3-540-44816-0_31

[B30] Scheme E. Englehart K. (2011). Electromyogram pattern recognition for control of powered upper-limb prostheses: state of the art and challenges for clinical use. J. Rehabil. Res. Dev. 48, 643–660. 10.1682/JRRD.2010.09.0177 21938652

[B31] Scheme E. J. Hudgins B. S. Englehart K. B. (2013). Confidence-based rejection for improved pattern recognition myoelectric control. IEEE Trans. Biomed. Eng. 60, 1563–1570. 10.1109/TBME.2013.2238939 23322756

[B32] Segil J. Kaliki R. Uellendahl J. Weir R. F. (2020). A myoelectric postural control algorithm for persons with transradial amputation: a consideration of clinical readiness. IEEE Robot. Autom. Mag. 27, 77–86. 10.1109/MRA.2019.2949688 32494115 PMC7269158

[B33] Segil J. L. Weir R. F. (2015). Novel postural control algorithm for control of multifunctional myoelectric prosthetic hands. J. Rehabil. Res. Dev. 52, 449–466. 10.1682/JRRD.2014.05.0134 26348320 PMC4666529

[B34] Sensinger J. W. Lock B. A. Kuiken T. A. (2009). Adaptive pattern recognition of myoelectric signals: exploration of conceptual framework and practical algorithms. IEEE Trans. Neural Syst. Rehabil. Eng. 17, 270–278. 10.1109/TNSRE.2009.2023282 19497834 PMC3025709

[B35] Settles B. (2009). Active Learning Literature Survey. Technical report, University of Wisconsin-Madison Department of Computer Sciences.

[B36] Shannon C. E. (1948). A mathematical theory of communication. Bell Syst. Techn. J. 27, 379–423. 10.1002/j.1538-7305.1948.tb01338.x30854411

[B37] Vidovic M. M.-C. Hwang H.-J. Amsüss S. Hahne J. M. Farina D. Müller K.-R. (2015a). Improving the robustness of myoelectric pattern recognition for upper limb prostheses by covariate shift adaptation. IEEE Trans. Neural Syst. Rehabil. Eng. 24, 961–970. 10.1109/TNSRE.2015.2492619 26513794

[B38] Vidovic M. M.-C. Hwang H.-J. Amsüss S. Hahne J. M. Farina D. Müller K.-R. (2016b). Improving the robustness of myoelectric pattern recognition for upper limb prostheses by covariate shift adaptation. IEEE Trans. Neural Syst. Rehabil. Eng. 24, 961–970. 26513794 10.1109/TNSRE.2015.2492619

[B39] Zhai X. Jelfs B. Chan R. H. Tin C. (2017). Self-recalibrating surface EMG pattern recognition for neuroprosthesis control based on convolutional neural network. Front. Neurosci. 11:379. 10.3389/fnins.2017.00379 28744189 PMC5504564

